# Gr1^−/low^CD11b^−/low^MHCII^+^ myeloid cells boost T cell anti‐tumor efficacy

**DOI:** 10.1002/JLB.5A0717-276RR

**Published:** 2018-07-09

**Authors:** Kyle K. Payne, Hussein F. Aqbi, Savannah E. Butler, Laura Graham, Rebecca C. Keim, Wen Wan, Michael O. Idowu, Harry D. Bear, Xiang‐Yang Wang, Masoud H. Manjili

**Affiliations:** ^1^ Department of Microbiology & Immunology Virginia Commonwealth University School of Medicine Richmond Virginia USA; ^2^ Massey Cancer Center Virginia Commonwealth University School of Medicine Richmond Virginia USA; ^3^ Department of Immunology Moffitt Cancer Center Tampa Florida USA; ^4^ Department of Surgery Virginia Commonwealth University School of Medicine Richmond Virginia USA; ^5^ Department of Biostatistics Virginia Commonwealth University School of Medicine Richmond Virginia USA; ^6^ Department of Human & Molecular Genetics Virginia Commonwealth University School of Medicine Richmond Virginia USA; ^7^ Department of Pathology Virginia Commonwealth University School of Medicine Richmond Virginia USA; ^8^ VCU Institute of Molecular Medicine Virginia Commonwealth University School of Medicine Richmond Virginia USA

**Keywords:** adoptive immunotherapy, Ag presenting cells, breast cancer, cancer vaccine, myeloid‐derived suppressor cells

## Abstract

Conventional APCs that express MHC class II (MHCII) and co‐stimulatory molecules include dendritic cells (DCs) and macrophages. Beyond these conventional APCs, immune stimulatory cells have been more recently shown to extend to a class of atypical APCs, composed of mast cells, basophils, and eosinophils. Here, we describe a unique type of APC, Gr1^−/low^CD11b^−/low^ cells with a granularity and size characteristic of myeloid cells and with the ability to present Ag for crosspresentation. These cells constitutively express MHCII and the costimulatory molecules, CD80, CD86, and CD40. They do not express pan markers of myeloid DCs (CD11c), plasmacytoid DCs (Ly6C), or macrophages (F4/80), and their frequency is inversely correlated with myeloid‐derived suppressor cells (MDSCs) in tumor‐bearing mice. Among splenocytes, they are more abundant than DCs and macrophages, and they exhibit antitumor immune stimulatory function at a steady state without further activation, ex vivo. They are also found within the tumor bed where they retain their immune stimulatory function. Our findings suggest the use of these novel APCs in additional preclinical studies to further investigate their utility in APC‐based cancer immunotherapies.

## Abstract 2

A new class of antigen presenting cells that are able to boost anti‐tumor immune responses is described.

**Abbreviations**
AIT: adoptive immunotherapy
DC: Dendritic cell
ILC: innate lymphoid cell
MDSC: myeloid‐derived suppressor cell

## 1 INTRODUCTION

Dendritic cells (DCs) play a central role in inducing immune responses against infectious diseases and cancer. However, their efficacy as a cell‐based vaccine is limited despite continued optimization of various vaccination parameters. This is in part due to the host‐derived immune suppressive cells such as myeloid‐derived suppressor cells (MDSCs). The accumulation of MDSCs hinders protective immune responses to cancer and infectious diseases such as tuberculosis,^1^, ^2^ AIDS,^3^, ^4^, ^5^ hepatitis C,^6^, ^7^ hepatitis B,^8^, ^9^ pneumonia,^10^, ^11^ and *Staphylococcus aurous* infection.^12^ Importantly, an elevation of MDSCs is associated with a reduced efficacy of vaccines.^13^, ^14^ In addition, the generation of monocyte‐derived DCs or bone marrow‐derived DCs requires extensive ex vivo culturing, conceivably hampering the immunogenicity of the vaccine. Recent studies, therefore, have focused on vaccines that make use of primary DCs.^15^ For instance, Sipuleucel‐T is the only FDA‐approved therapeutic vaccine for metastatic prostate cancer.^16^ The vaccine uses readily isolated circulating DCs cultured with prostate tumor Ag and GM‐CSF. However, circulating DCs are very rare and tumor‐induced immune suppressive cells, such as MDSCs, limit their efficacy in inducing a sustained antitumor immune response. Therefore, there is an urgent need to identify a new class of APC that are highly efficient in orchestrating profound antitumor immunity to facilitate the development of a new class of cell‐based cancer vaccines.

In recent years, there has been a rapid increase in our understanding of the biology of cells with APC characteristics, namely the ability to activate T cells. For instance, mouse neutrophils can induce Th1 and Th17 responses^17^, ^18^ and tumor‐associated neutrophils have been demonstrated to stimulate T cell responses in early‐stage human lung cancer.^19^ A recent review discusses a number of atypical APCs including mast cells, basophils, eosinophils, and innate lymphoid cells (ILC).^20^, ^21^ However, these APCs are rare in the circulation and their maintenance of effective antitumor immune responses is likely to be inhibited due to high frequencies of MDSCs in locations of T cell priming. Very recently, it was reported that activated NKT cells decrease the frequency and immunosuppressive activity of MDSCs in tumor‐bearing mice.^22^ In an animal model, activated NKT cells converted MDSCs into immunogenic APCs.^23^ Using peripheral blood mononuclear cells (PBMC) of patients with early stage breast cancer, we also demonstrated that conversion of MDSCs to CD33^+^CD11b^−/low^HLA‐DR^+^ APCs, in vitro, was associated with an increased frequency of CD25+ NKT cells in reprogrammed immune cells.^24^

In an effort to understand this MDSC‐APC axis during the application of adoptive immunotherapy (AIT) to treat breast cancer, we identified a class of Gr1^−/low^CD11b^−/low^ MHCII+ APCs. These cells retain their immune stimulatory function during tumor progression and are inversely correlated to the frequency of splenic and tumor‐infiltrating MDSCs. Importantly, we identified the presence of these cells in nonpathological conditions, whereupon we confirmed their ability to cross‐present Ag to stimulate T cells. Therefore, these APCs offer a potentially novel APC‐based vaccine for cancer therapy.

## 2 MATERIALS AND METHODS

### 2.1 Mouse model

FVBN202 transgenic female mice (The Jackson Laboratory; Bar Harbor, ME) were used between 8 and 12 weeks of age throughout these experiments. These mice overexpress a nonmutated, nonactivated rat neu transgene under the regulation of the mouse mammary tumor virus promoter.^25^ These mice develop premalignant mammary hyperplasia similar to ductal carcinoma in situ prior to the development of spontaneous carcinoma.^26^ Premalignant events in FVBN202 mice include the accumulation of endogenous MDSCs.^26^ These studies have been reviewed and approved by the Institutional Animal Care and Use Committee at Virginia Commonwealth University.

### 2.2 Tumor cell lines

The neu overexpressing mouse mammary carcinoma (MMC) cell line was established from a spontaneous mammary tumor harvested from FVBN202 mice. Tumor cells were maintained in RPMI 1640 supplemented with 10% FBS.

### 2.3 Ex vivo reprogramming and expansion of splenocytes

Reprogramming of tumor‐sensitized immune cells was performed as previously described by our group.^5^ Briefly, FVBN202 transgenic mice were inoculated in the mammary fat pad with 3 × 10^6^ MMC cells. Tumor growth was monitored by digital caliper, and tumor volumes were calculated by volume (*v*) = (*L* [length] × *W* [width]^2^)/2. As previously described,^11^ splenocytes were harvested 21–25 days after tumor challenge, when the tumor had reached ≥ 1000 mm.^3^ Splenocytes were then cultured in complete medium (RPMI 1640 supplemented with 10 % FBS, l‐glutamine (2 mM), 100 U/ml penicillin, and 100 μg/ml Streptomycin) and were stimulated with Bryostatin 1 (2 nM; Sigma, Saint Louis, MO), Ionomycin (1 μM; Calbiochem, San Diego, CA), and 80 U/ml/10^6^ cells of IL‐2 (Peprotech) for 16–18 h.^24^, ^27^ Lymphocytes were then washed thrice and cultured at 10^6^ cells/ml in complete medium with IL‐7 and IL‐15 (20 ng/ml of each cytokine, Peprotech, Rocky Hill, NJ). After 24 h, 20 U/ml of IL‐2 was added to the complete medium. The following day, the cells were washed and cultured at 10^6^ cells/ml in complete medium with 40 U/ml of IL‐2. After 48 h, cells were washed and cultured at 10^6^ cells/ml in complete medium with 40 U/ml of IL‐2. After 24 h, lymphocytes were again washed and cultured at 10^6^ cells/ml in complete medium with 40 U/ml of IL‐2. Lymphocytes were harvested 24 h later on the sixth day and were then either used in AIT or analyzed ex vivo. Reprogramming of splenocytes consistently yielded 5‐fold expansion with greater than 40% memory T cells and 35% CD25+ NKT cells.^27^

### 2.4 Adoptive cellular therapy

Twenty‐four hours prior to AIT, FVBN202 mice were injected i.p. with CYP (100 mg/kg) to induce lymphopenia. Approximately 18 h later FVBN202 mice were challenged i.v. with MMC cells (1 × 10^5^). Mice then received adoptive transfer of reprogrammed splenocytes i.v. at a dose of 70 × 10^6^/mouse later the same day (AIT), or remained untreated (Control). The study end‐point and euthanasia occurred when the animals were considered moribund upon losing 10–20% of their initial body weight due to disease progression.

### 2.5 Characterization of splenocytes and tumor‐infiltrating leukocytes

Spleens and metastases of tumor‐bearing FVBN202 mice were harvested when the animals became moribund, and were then homogenized into a single cell suspension as described previously^28^ and below; single cell suspensions were then characterized using flow cytometry. Reagents used for flow cytometry: anti‐CD16/32 Ab (93); FITC‐CD11b (M1/70); PE‐GR‐1 (RB6‐8C5); PE‐CD11c (N418); PE‐F4/80 (BM8); PE‐CD25 (3C7); Allophycocyanin‐CD49b (DX5); Allophycocyanin‐Annexin V; Alexa Fluor 647‐I‐Aq (KH116); Alexa Fluor 700 Ly‐6G (1A8); PercP/CY5.5‐CD86 (GL‐1); PercP/CY5.5‐Rat IgG2a, k Isotype Control (RTK2758); PE‐Dazzle‐CD80 (16‐10A1); PE‐Dazzle‐Armenian Hamster IgG Isotype Control (HTK888); PE/CY7‐CD40 (3/23); PE/CY7‐Rat IgG2a, k Isotype Control (RTK2758); Brilliant Violet 510 Ly‐6C (HK1.4); Brilliant Violet 605‐CD45 (30‐F11); BV421‐CD20 (SA275A11); BV711‐Ly6C (HK1.4); BV510‐CD11b (M1/70); and BV785‐CD86 (GL‐1), all of which were purchased from Biolegend (San Diego, CA). BD Horizon V450‐Annexin V and BUV395‐CD3 (SK7) were purchased from BD Biosciences (Franklin Lakes, NJ). Propidium Iodide (PI) was purchased from Sigma. (All reagents were used at the manufacturer's recommended concentration. Cellular staining was performed as previously described by our group.^24^ Multicolor data acquisition was performed using a LSRFortessa X‐20 (BD Biosciences) and a ImageStreamX Mark II Imaging Flow Cytometer (Millipore Sigma, Billaerica, MA). Data was analyzed using FCS Express v4.07 and v5.0 (De Novo Software; Glendale, CA).

### 2.6 Sorting of myeloid cells by FACS

Splenocytes were stained for surface expression of CD11b and Gr1 as described above. Isolated cells were gated on the myeloid cell population based on their inherent light scattering properties^29^ thereby excluding cells of lymphoid origin. Gr1^−/low^CD11b^−/low^ myeloid cells from the Control and AIT groups were then sorted into independent populations using a FACSAria (BD Biosciences) as previously described.^30^ Purity of sorted cells was consistently greater than 90%.

### 2.7 IFN‐γ ELISA

Splenocytes from the Control and AIT groups were independently cultured in serum‐free RPMI 1640 in order to enrich for nonadherent cells.^31^ After 2 h, nonadherent lymphocytes were cultured in complete medium with irradiated MMC cells (140 Gy) at a 10:1 ratio, and with or without sorted Gr1^−/low^CD11b^−/low^ myeloid cells at a 2:1 ratio, for 20 h. Also, sorted Gr1^−/low^CD11b^−/low^ cells or bone marrow‐derived DCs were pulsed with recombinant rat Neu extracellular domain (50 ug/ml) in the presence of GM‐CSF (20 ng/ml) for 24 h, washed of free protein, and co‐cultured with tumor‐sensitized, reprogrammed T cells (1:3) for 20 h. Irradiated MMC (140 Gy) were used as positive target for tumor‐sensitized reprogrammed T cells (1:10 ratio). Supernatants were then collected and stored at −80°C until assayed. IFN‐γ was detected in the supernatant using a Mouse IFN‐γ ELISA kit (BD Biosciences), according to the manufacturer's protocol.

### 2.8 In vitro Ag uptake

Splenocytes (10^6^ cells/ml) of naïve FVBN202 mice were pulsed with 50 ug/ml Alexa Fluor 488 (AF488)‐conjugated ovalbumin (ThermoFisher Scientific) in RPMI1640 supplemented with 10% FBS for 5 or 16 h. Cells were then washed and stained for FVS, CD11c, CD11b, Gr1. Gated FVS‐ viable cells were subgated for CD11c+ DCs or Gr1^−/low^CD11b^−/low^ myeloid cells, and analyzed for Alexa Fluor 488 as a reporter of OVA internalization.

### 2.9 Cytotoxicity assay

Antitumor efficacy of T cells was determined in a cytotoxicity assay, in vitro, using flow cytometry as previously described by our group^32^ with minor modifications. The ex vivo expanded tumor reactive T cells were cultured in complete medium with MMC cells (10:1 E:T ratio) in the presence or absence of sorted Gr1^−/low^CD11b^−/low^ cells at a 5:1 ratio (five T cells vs. one APCs), for 48 h. Cells were collected and stained with Annexin V, PI, anti‐CD45 and anti‐Neu Abs immediately prior to flow cytometry acquisition.

### 2.10 Isolation of tumor‐infiltrating leukocytes from lung metastases

Lungs were harvested from the Control and AIT groups after animals became moribund. Metastatic lesions were individually excised from the residual lung tissue, and were minced and digested in Trypsin‐EDTA (0.25%; Life Technologies) overnight at 4°C. The following day, the suspension was incubated at 37°C for 30 min, followed by gentle tissue homogenization to create a cellular suspension. The cell suspension was then washed twice with RPMI supplemented with 10% FBS. Residual red blood cells were then lysed using ACK lysing buffer, followed by an additional wash with RPMI 10% FBS. 10^6^ cells of the suspension were then stained for surface molecules as described above. All analysis was performed by gating on viable leukocytes (CD45+ Annexin V^−^).

### 2.11 Statistical analysis

Outcomes are summarized by basic descriptive statistics such as mean and sem; differences between groups are illustrated using graphical data presented as mean ± sem. Statistical comparisons between groups were made using one‐tailed and two‐tailed Student's *t‐*test per the specific hypothesis. Time to death in the in vivo survival studies was calculated from baseline to the date of death. Mice were euthanized when they had a weight loss of ≥10%. Kaplan–Meier curves and log‐rank tests are used to illustrate time to death and to test the difference between each group. A *P*‐value ≤ 0.05 was considered statistically significant.

## 3 RESULTS

### 3.1 Gr1^−/low^CD11b^−/low^ cells demonstrate characteristics of professional APCs

**Figure 1:**
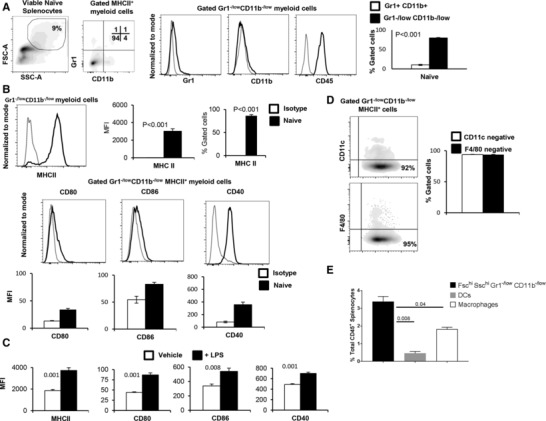
**Splenic Gr1^−/low^CD11b^−/low^ cells show characteristics of APCs. (**A) Splenocytes of naïve FVBN202 mice (*n* = 3) were gated within the myeloid cell region based on forward‐scatter and side‐scatter, and were analyzed for the expression of Gr1 and CD11b. The proportion of the splenic Gr1^−/low^CD11b^−/low^ myeloid cells and Gr1^+^CD11b^+^ myeloid cells was determined. (B) Gated Gr1^−/low^CD11b^−/low^ cells were analyzed for the expression of MHC class II (MHCII). Gated Gr1^−/low^CD11b^−/low^MHCII^+^ cells were also analyzed for the expression of the co‐stimulatory molecules, CD80, CD86, and CD40. Mean fluorescence intensity (MFI) of the co‐stimulatory molecules showed a significant shift compared with isotype control. (C) Sorted Gr1^−/low^CD11b^−/low^MHCII^+^ cells were cultured in the absence (–LPS) or presence of LPS (+LPS, 1μg/ml) for 24 h. Gated Gr1^−/low^CD11b^−/low^MHCII^+^ cells were analyzed for the expression of MHCII as well as CD80, CD86, or CD40. MFI was calculated after the subtraction of isotype control. (D) Gated Gr1^−/low^CD11b^−/low^MHCII^+^ cells were analyzed for the expression of CD11c or F4/80. (E) Percent total frequency of MHCII+ Gr1^−/low^CD11b^−/low^, DCs and macrophages in the spleen. Data represent mean ± sem. Data are representative of at least 3 independent experiments

Antitumor immune responses are often corrupted in tumor bearing hosts due to pathological emergency myelopoiesis, which leads to the accumulation of MDSCs in secondary lymphoid organs and tumor beds.^33^, ^34^ However, it has been reported that lymphoid effectors, namely NKT cells, functionally alter MDSC function by promoting an immunostimulatory, rather than suppressive, phenotype in the context of antitumor immunity.^24^, ^27^ Therefore, we sought to gain an understanding of the biology of myeloid cells under nonpathological conditions in order to appreciate their functional plasticity. First, we observed that the splenic Fsc^hi^ Ssc^hi^ myeloid cell compartment of naïve mice was dominated by a population of Gr1^−/low^CD11b^−/low^ cells (Fig. 1A, right panel; *P* = 0.00002), which were of hematopoietic origin. Furthermore, these Gr1^−/low^CD11b^−/low^ cells demonstrated expression of MHC class II (MHCII; *P* = 0.0002) and the co‐stimulatory molecules, CD80 (*P* = 0.001), CD86 (*P* = 0.009), and CD40 (*P* = 0.0003), as shown in Fig. 1B. LPS stimulation induced the maturation of Gr1^−/low^CD11b^−/low^ cells (Fig. 1C) by up‐regulating the expression of MHCII (MFI: 1851 vs. 3732, *P* = 0.001), CD80 (MFI: 44 vs. 87, *P* = 0.001), CD86 (MFI: 338 vs. 541, *P* = 0.008) and CD40 (MFI: 488 vs. 800, *P* = 0.001). Despite displaying such classical characteristics of APCs, Gr1^−/low^CD11b^−/low^ cells did not express pan markers of DCs, CD11c, or macrophages, F4/80 (Fig. 1D). Importantly, however, these Fsc^hi^ Ssc^hi^ Gr1^−/low^CD11b^−/low^ myeloid cells possess a similar size and granularity, and express similar levels of MHCII as well as costimulatory molecules to total splenic macrophages and dendritic cells (Supplementary Fig. 1). The total frequency of Fsc^hi^ Ssc^hi^ Gr1^−/low^CD11b^−/low^ APCs was significantly higher than all DCs and macrophages in the spleen (Fig. 1E, *P* = 0.008 and *P* = 0.04, respectively). Additionally, morphological studies of these cells using Diff‐Quick staining demonstrated the presence of both monoblast‐like (large cells), and lymphocyte‐like (small cells) within the Fsc^hi^ Ssc^hi^ Gr1^−/low^CD11b^−/low^ gate (Supplementary Fig. 2).

**Figure 2:**
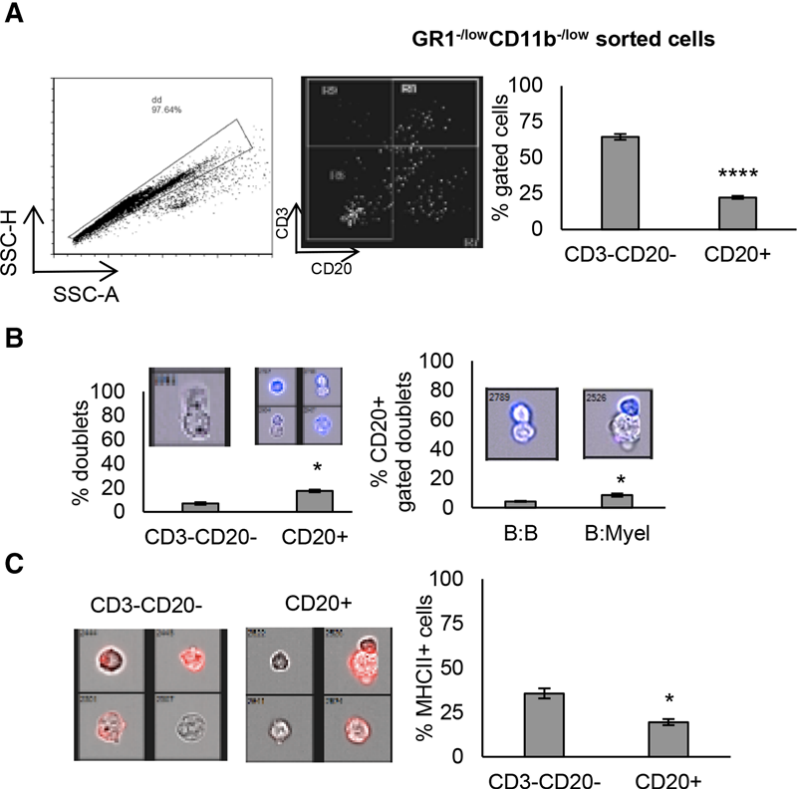
**GR1^−/low^CD11b^−/low^ cells contain myeloid cells and B cells**. GR1^−/low^CD11b^−/low^ cells within the myeloid region of the scatter plot were sorted and analyzed via Image Stream. (A) After excluding doublets, cells were analyzed for CD3 and CD20 expression to determine if T and B cells were still falling within the myeloid gate. (B) Hundred images/events from the CD3^−^CD20^−^ and CD20^+^ populations were analyzed for doublets by inspecting each image manually. Also, doublets within CD20+ cells were analyzed based on morphology showing B cell:B cells (B:B) or B cells:Myeloid cells (B:Myel) interactions. (C) MHCII (red) expression on CD3^−^CD20^−^ and CD20^+^ populations. Data represent mean ± sem of triplicate experiments

Given that Diff‐Quick staining revealed the presence of lymphocyte‐like cells among sorted Gr1^−/low^CD11b^−/low^ cells from naïve mice, we sought to further determine the phenotype and frequency of these cells within the sorted population. We found that a majority of gated Gr1^−/low^CD11b^−/low^ cells lacked expression of lineage markers for T or B cells (CD3^−^CD20^−^), although 22% of cells included CD20^+^ B cells (Fig. 2A). We then hypothesized that the presence of residual B‐cells in the Fsc^hi^ Scc^hi^ myeloid region was due to cell‐to‐cell interactions between B cells and myeloid cells. To investigate this, ImageStreamX analysis was performed. The total events were analyzed for percentage of events that had two cells contained in one event by observing each event manually. The number of doublets containing the CD20^+^ population was significantly higher in comparison to the CD3^−^CD20^−^ doublets (Fig. 2B, left and middle panels, 7% vs. 17.5%). Among CD20^+^ B cells in this population, the majority of cell‐to‐cell contacts were shown to be B cell:myeloid cell interactions (B:Myel), rather than B cell:B cell (B:B) interactions (Fig. 2B, right panel, 9% vs. 4%). We then determined the source of MHCII expression among these interacting cells. As can be seen in Fig. 2C, myeloid cells (CD20^–^CD3^–^) had significantly higher percent of MHCII expression compared to CD20^+^ cells. Taken together, our data suggest the presence of a unique lineage of myeloid‐derived APC, which demonstrates characteristics of classical APC:B cell interactions in naïve mice.^35^, ^36^

### 3.2 Gr1^−/low^CD11b^−/low^ MHCII^+^ cells are heterogeneous populations that are both lineage committed and noncommitted

**Figure 3:**
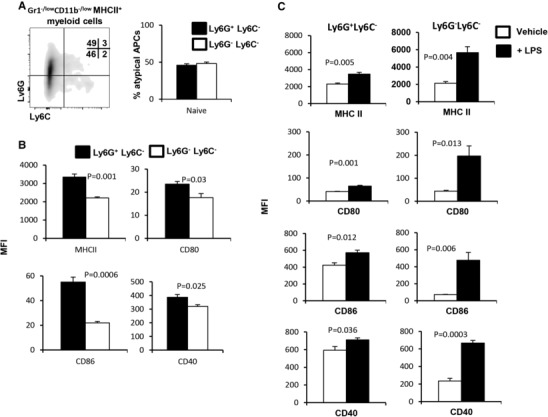
**Gr1^−/low^CD11b^−/low^ MHCII^+^ myeloid cells contain Ly6G^+^Ly6C^−^ and Ly6G^−^Ly6C^−^ subsets. (**A) Splenocytes of naïve FVBN202 mice (*n* = 3) were gated within the myeloid cell region and expression of Ly6G and Ly6C was determined on gated Gr1^−/low^CD11b^−/low^ MHCII^+^ cells. (B) Expression of MHCII and co‐stimulatory molecules was determined on gated MHCII^+^Ly6G^+^Ly6C^−^ and MHCII^+^Ly6G^−^Ly6C^−^ cells. (C) Expression of MHCII and co‐stimulatory molecules was determined on gated Ly6G^+^Ly6C^−^ or Ly6G^−^Ly6C^−^ subsets after 24 h stimulation in the absence (–LPS) or presence of LPS (+LPS, 1μg/ml). MFI were calculated after subtraction of isotype control. Data represent mean ± sem. Data are representative of at least 3 independent experiments

To further unravel the biology of Gr1^−/low^CD11b^−/low^ MHCII^+^ cells, we found that approximately 50% of these cells expressed Ly6G, indicative of a commitment to the granulocyte lineage, while the remainder of this population was negative for both Ly6G and Ly6C (Fig. 3A). Accordingly, the Ly6G^+^Ly6C^−^ subset displayed a more mature phenotype than the Ly6G^−^Ly6C^−^ subset, expressing significantly higher levels of MHCII (*P* = 0.001), CD80 (*P* = 0.03), CD86 (*P* = 0.0006), and CD40 (*P* = 0.025; Fig. 2B). As the Ly6G^−^Ly6C^−^ subset did not demonstrate a specific myeloid‐cell lineage commitment by any parameter that we tested, we hypothesized that this population would respond more robustly to activating stimuli due to a presumed lack of maturity. Indeed, as shown in Fig. 3C, the Ly6G^−^Ly6C^−^ subset showed a stronger response to LPS stimulation when compared to vehicle treatment than the Ly6G^+^Ly6C^−^ subset. This suggests that under nonpathological conditions there exists a population of both lineage committed and noncommitted splenic Gr1^−/low^CD11b^−/low^ MHCII^+^ cells, which possess the potential to perform professional Ag‐presenting cellular functions.

### 3.3 Adoptive immunotherapy modulates Gr1^−/low^CD11b^−/low^ APCs

It has been reported that activated NKT cells can convert MDSCs into immune‐stimulatory APCs.^22^, ^23^ We have reported that reprogrammed lymphocytes containing CD25+ NKT cells can induce maturation of human CD33^+^CD11b^+^HLA‐DR^−^ MDSCs into stimulatory CD33^+^CD11b^−/low^HLA‐DR^+^ APCs, in vitro.^24^, ^27^ Given the inverse correlation between Gr1^+^CD11b^+^ cells and Gr1^−/low^CD11b^−/low^ APCs in naïve mice (Fig. 1A), we sought to determine the impact of tumor burden as well as AIT, containing conventional tumor‐specific T cells and CD25+ NKT cells, on the modulation of Gr1^−/low^CD11b^−/low^ APCs, in vivo. FVBN202 mice were challenged i.v. with Neu‐overexpressing MMC tumor cells, and then either remained untreated (control) or were subjected to an adoptive transfer of tumor‐sensitized reprogrammed T cells and NKT cells.^32^ Animals were sacrificed upon disease progression culminating in metastases in the lung. As shown in Fig. 4A, AIT significantly prolonged animal survival (*P* = 0.015). Such antitumor protection was associated with modulation of the myeloid cell compartment, resulting in a significantly increased frequency of Gr1^−/low^CD11b^−/low^ APCs (Fig. 4B, 56% vs. 38%); the frequency of these cells dominated Gr1^+^CD11b^+^ MDSCs in the AIT group compared to the control group (Fig. 4B, 56% vs. 33%), even at equally advanced stages of tumor progression. Unlike naive mice and AIT recipients, the myeloid cellular compartment of the untreated control group mainly consisted of MDSCs (Fig. 4B, *P* = 0.03). The emergence of Gr1^−/low^CD11b^−/low^ APCs in the animals treated with AIT was associated with a significantly increased frequency of splenic CD25^+^ NKT cells compared with the control group (Supplementary Fig. 3, *P* = 0.037). Further analyses showed similar levels of MHCII expression (MFI and % gated) in both groups, though those treated with AIT had a significantly higher frequency of Gr1^−/low^CD11b^−/low^MHCII^+^ APCs among all splenocytes (Fig. 4C, *P* = 0.001). AIT also resulted in the up‐regulation of CD86 (Fig. 4D, MFI: 32 vs. 66, *P* = 0.01) and down‐regulation of CD40 (Fig. 4D, 616 vs. 278, *P* = 0.001) on Gr1^−/low^CD11b^−/low^ cells. In fact, AIT restored the frequency of Gr1^−/low^CD11b^−/low^MHCII^+^ APCs and the expression of CD40 to the levels similar to those in naive mice, though CD86 expression was uniquely up‐regulated following AIT (Supplementary Fig. 4A). AIT also resulted in a significantly increased frequency of splenic CD11c^+^ DCs and F4/80^+^ macrophages (Supplementary Fig. 4B, *P* = 0.001 and *P* = 0.018, respectively). In order to determine whether Gr1^−/low^CD11b^−/low^ APCs of the control and AIT groups had the capacity to respond to inflammatory stimuli and undergo maturation, LPS stimulation was performed in vitro. While LPS stimulation resulted in similar trends for both groups, as shown in Fig. 4E, tumor burden with or without AIT resulted in a unique pattern of maturation; we observed that Gr1^−/low^CD11b^−/low^ cells of the AIT group increased the expression of CD86 (MFI: 360 vs. 667, *P* = 0.022) and CD40 (MFI: 662 vs. 902, *P* = 0.023) whereas those of the control group increased the expression of MHCII (MFI: 2200 vs. 5647, *P* = 0.02), CD80 (MFI: 53 vs. 107, *P* = 0.053), and CD86 (MFI: 282 vs. 525, *P* = 0.042).

Figure 4
**Gr1^−/low^CD11b^−/low^ myeloid cells are modulated during tumor challenge or AIT. (**A) Kaplan–Meier analysis of survival in FVBN202 mice that were injected with 10^5^ MMC cells i.v.; animals were sacrificed when they became moribund due to lung metastases. (B) Splenocytes of the control and AIT groups were analyzed by flow cytometry after staining with fluorescently labeled anti‐Gr1 and anti‐CD11b Abs. Data show the frequency of the splenic Gr1^−/low^CD11b^−/low^ cells and MDSCs in the control and AIT groups. (C) Frequency and expression levels of MHCII were determined on gated Gr1^−/low^CD11b^−/low^MHCII^+^ cells of the AIT and control groups. (D) Gated Gr1–/low CD11b–/low cells were analyzed for the expression of co‐stimulatory molecules in the spleens of the AIT and control groups. (E) Gr1^−/low^CD11b^−/low^ cells were sorted from the spleens of the AIT and control groups and cultured for 24 h in the presence or absence of LPS (+LPS and –LPS). Gated cells were then analyzed for the expression of MHCII and co‐stimulatory molecules. Data represent mean ± sem of triplicate experiments

**Figure:**
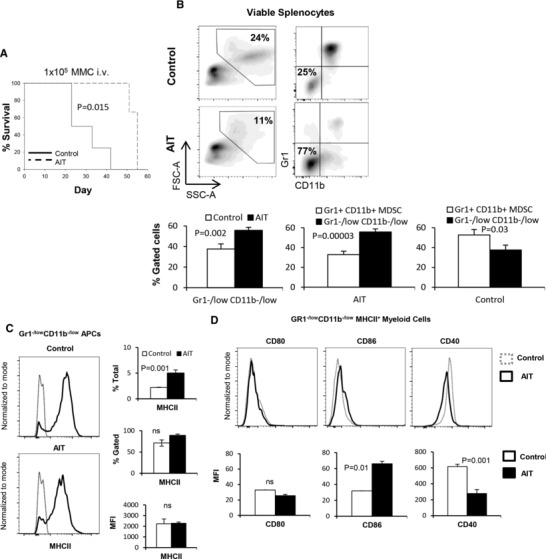

**Figure:**
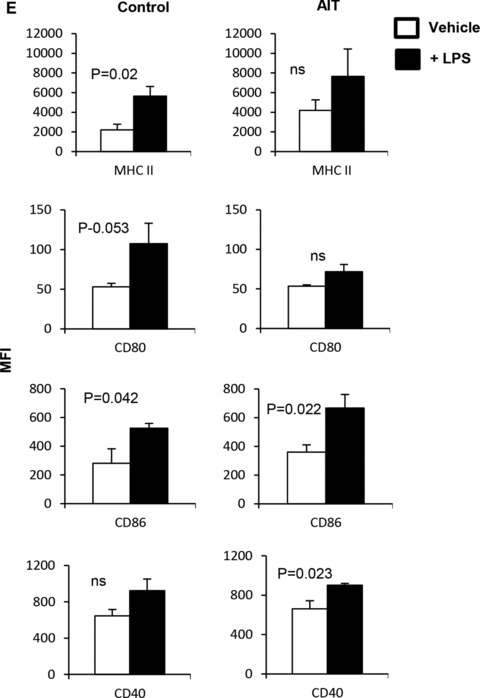

As the Ly6G^+^Ly6C^−^ subset had a higher expression of co‐stimulatory molecules than the Ly6G^−^Ly6C^−^ subset in naïve mice (Fig. 3), we sought to determine whether this trend was also present during tumor burden or following AIT. Subset analysis of Gr1^−/low^CD11b^−/low^ APCs showed the emergence of a Ly6G^+^Ly6C^−^ cell population in tumor‐bearing mice that received AIT when compared with the control group (Fig. 5A, 35% vs. 7%). Unlike untreated tumor‐bearing mice, animals receiving AIT showed a similar trend with naïve mice in regards to the frequency of Ly6G^+^Ly6C^−^ myeloid cells (Supplementary Fig. 5). Whereas both subsets showed comparable levels of the expression of MHCII, CD80, and CD40 in the control and AIT groups, the Ly6G^+^Ly6C^−^ subset exhibited a significantly higher level of CD86 expression (Fig. 5B, Control, MFI: 17 vs. 27; AIT, MFI: 16 vs. 41). As expected, the mature Ly6G^+^Ly6C^−^ subset did not result in an increase in the expression of CD86 following LPS stimulation. However the Ly6G^−^Ly6C^−^ subset in the control group and in the AIT group did experience increases in the expression of CD80 (Fig. 4C, *P* = 0.042 and *P* = 0.058) and CD86 (Fig. 4C, *P* = 0.004 and *P* = 0.058). The Ly6G^−^Ly6C^−^ subset within control mice also increased the expression of MHCII (*P* = 0.021) and CD40 (*P* = 0.05) following LPS stimulation. Therefore, these data suggest that AIT rescues the myeloid compartment of tumor‐bearing animals by promoting the maturation of myeloid cells to the frequency and functional potential observed in naïve mice.

Figure 5
**Tumor burden or AIT modulates Gr1^−/low^CD11b^−/low^ myeloid cells. (**A) Splenocytes of FVBN202 mice bearing metastatic tumor in the lung without treatment (Control) or after AIT (AIT) were subjected to analysis by flow cytometry. (A) Comparative analysis of Ly6G^+^Ly6C^−^ and Ly6G^−^Ly6C^−^ subsets among gated APCs of control and AIT groups. (B) Expression of MHCII and co‐stimulatory molecules on Ly6G^+^Ly6C^−^ and Ly6G^−^Ly6C^−^ subsets in gated APCs of control and AIT groups. Gated cells were then analyzed for the expression of MHCII and co‐stimulatory molecules. (C) Gr1^−/low^CD11b^−/low^ APCs were sorted from the spleens of the AIT and control groups, and cultured for 24 h in the presence or absence of LPS (+LPS and –LPS). Data represent mean ± sem of triplicate experiments

**Figure:**
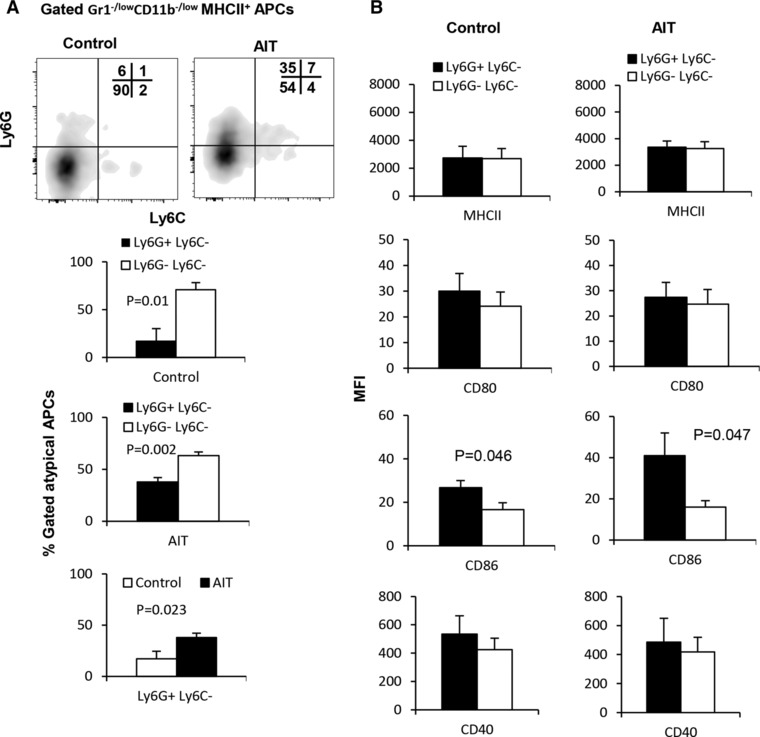

**Figure:**
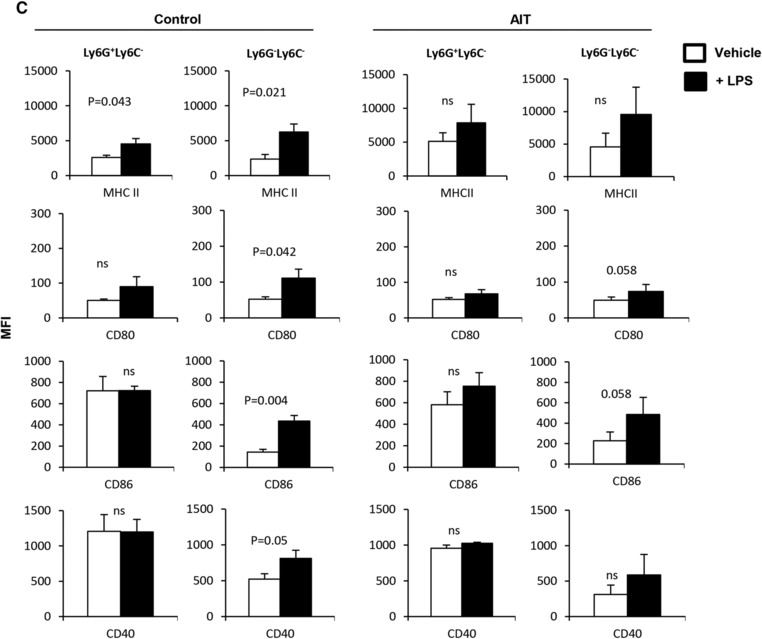

### 3.4 Gr1^−/low^CD11b^−/low^ Ly6G^+^Ly6C^−^ APCs are present within the tumor bed

To determine whether Gr1^−/low^CD11b^−/low^ APCs are present in the tumor bed, tumor lesions of both the AIT and control groups were analyzed when animals were euthanized due to tumor progression with similar tumor burden. As in the spleen, we again found that Gr1^−/low^CD11b^−/low^ cells dominated the tumor‐infiltrating Fsc^hi^ Ssc^hi^ myeloid cell compartment within the AIT group, where they demonstrated a greater than 3‐fold increase in frequency over Gr1^+^CD11b^+^ MDSCs (Supplementary Fig. 6A, 14% vs. 46%, p = 0.016). Such differences were, again, not observed in the control group, These Gr1^−/low^CD11b^−/low^ APCs had similar pattern of maturation between the AIT and control groups to that of the spleen, as shown by comparable levels of the expression of MHCII, CD80, CD86, and CD40 (Supplementary Fig. 6B). Within the tumor bed, the Ly6G^+^Ly6C^−^ subset was clearly dominant within the AIT group (Supplemental Fig. 6C, 63% vs. 16%; *P* = 0.014). Whereas both subsets showed comparable levels of costimulatory molecule expression, the Ly6G^+^Ly6C^−^ subset demonstrated more robust MHCII expression at the tumor site of the AIT group (Supplementary Fig. 6D, MFI: 11984 vs. 4739, *P* = 0.026).

### 3.5 Gr1^−/low^CD11b^−/low^ APCs exhibit immune stimulatory function

**Figure 6:**
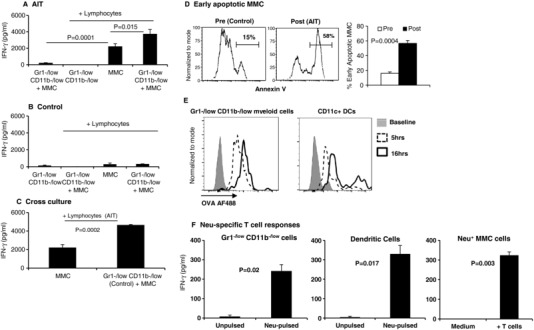
**Gr1^−/low^CD11b^−/low^ myeloid cells retain their immune stimulatory function during tumor burden and display characteristics of Ag‐presentation**. FACS sorted splenic Gr1^−/low^CD11b^−/low^ cells from (A) AIT recipients or (B) Control mice were co‐cultured without or with MMC (5:1) and without or with endogenous splenic lymphocytes (1:2) for 20 h; supernatant IFN‐γ concentration was determined by ELISA. (C) Lymphocytes of the AIT group were cultured with MMC in the presence or absence of sorted Gr1^−/low^CD11b^−/low^ cells of the control group. Data represent mean ± sem after subtracting background signal from control conditions. (D) Quantification of Annexin V+ early apoptotic MMC cells after culture with freshly isolated lymphocytes of tumor‐bearing control mice prior to the ex vivo re‐programming (Pre) or with re‐programmed lymphocytes used for AIT (Post). Data represent quadruplicate experiments. (E) Splenocytes (10^6^ cells/ml) of naïve FVBN202 mice were pulsed with 50 ug/ml Alexa Fluor 488 (AF488)‐conjugated ovalbumin in RPMI1640 supplemented with 10% FBS for 5 or 16 h. Unpulsed cells were used as control (Baseline). Gated FVS– viable cells were subgated for CD11c+ DCs or Gr1^−/low^CD11b^−/low^ cells, and analyzed for intensity of Alexa Fluor 488 as a marker of ovalbumin internalization. (F) Sorted Gr1^−/low^CD11b^−/low^ splenic cells or bone marrow‐derived CD11c+ DCs were pulsed with Neu ECD and cultured with tumor‐sensitized T cells. Irradiated MMC target cells were used as a positive control

In order to determine if Gr1^−/low^CD11b^−/low^ APCs possess immune stimulatory function during tumor burden and/or following AIT, splenic lymphocytes from the AIT and control group were independently cultured with MMC tumor cells in the presence or absence of sorted autologous Gr1^−/low^CD11b^−/low^ cells. As shown in Fig. 6A, lymphocytes derived from the AIT group released IFN‐γ in the presence of Neu^+^ MMC cells (*p* = 0.0001). Importantly, the IFN‐γ producing immune response to MMC was significantly boosted by autologous Gr1^−/low^CD11b^−/low^ APCs (Fig. 6A, *p* = 0.015). On the other hand, lymphocytes derived from the control group did not demonstrate significant IFN‐γ release in the presence of MMC; the addition of autologous Gr1^−/low^CD11b^−/low^ APCs did not enhance this response (Fig. 6B). In order to determine if Gr1^−/low^CD11b^−/low^ myeloid cells from the control group retained their immune stimulatory function, they were co‐cultured with tumor‐reactive T cells from the AIT group in the presence or absence of MMC. We hypothesized that T cell specific killing of MMC cells from the AIT group could facilitate cross presentation of tumor Ags by Gr1^−/low^CD11b^−/low^ APCs, resulting in the enhancement of the immune response. As shown in Fig. 6C, the presence of Gr1^−/low^CD11b^−/low^ APCs boosted tumor‐reactive IFN‐γ production by splenic T cells derived from the AIT group (*P* = 0.0002). This was associated with the induction of apoptosis in MMC by reprogrammed T cells that were used for AIT compared with those of the control group (Fig. 6D, *P* = 0.0004). To assess the possibility of Gr1^−/low^CD11b^−/low^ myeloid cells to potentially uptake and cross‐present Ag to T cells, we first pulsed these cells with ovalbumin conjugated to a fluorophore. As shown in Fig. 6E, Gr1^−/low^CD11b^−/low^ myeloid cells demonstrated the ability to uptake this protein, with increased fluorescence intensity over time. Although it appears these cells have a reduced efficiency to uptake this Ag compared to DCs, these data suggest that Gr1^−/low^CD11b^−/low^ myeloid cells may potentially function to cross‐present processed Ag to T cells.

**Figure 7:**
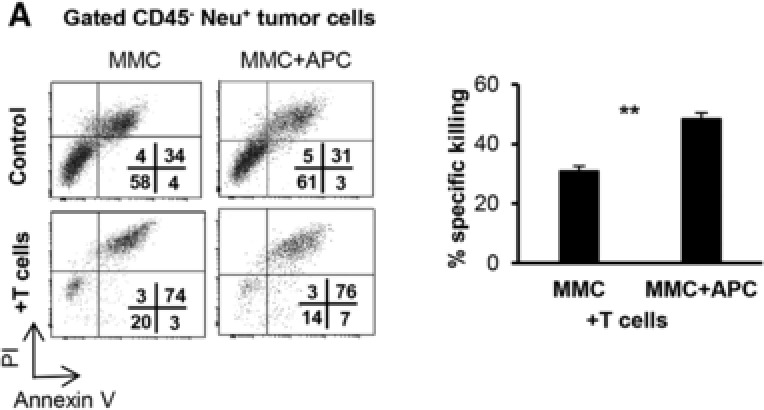
**Gr1^−/low^CD11b^−/low^ APCs boost antitumor function of T cells. (A)** Tumor‐reactive T cells derived and expanded from FVBN202 mice were co‐cultured with MMC (10:1 ratio) in the presence or absence of sorted Gr1^−/low^CD11b^−/low^ cells (5:1 ratio). Tumor cell cytotoxicity was determined on gated CD45‐Neu+ tumor cells using control tumor cells alone, or in the presence of sorted Gr1^−/low^CD11b^−/low^ APCs, T cells, or sorted Gr1^−/low^CD11b^−/low^ APCs and T cells. Percent increased apoptosis of tumor cells by T cells in the absence (MMC) or presence of Gr1^−/low^CD11b^−/low^ APCs (MMC+APC) was calculated by normalizing to the respective control. Data represent mean ± sem of triplicate experiments

Thus, to specifically determine if Gr1^−/low^CD11b^−/low^ myeloid cells could cross‐present Ag to provoke a T cell response, we sorted splenic Gr1^−/low^CD11b^−/low^ myeloid cells and pulsed them with recombinant Neu ECD protein, followed by a culturing period with tumor‐sensitized T cells. In fact, as can be seen in Fig. 6F, Gr1^−/low^CD11b^−/low^ myeloid cells were able to induce IFN‐γ production from tumor‐sensitized T cells only after they were pulsed with Neu ECD, suggesting these cells possess Ag‐processing and presentation functionality. Bone marrow‐derived DCs were used a positive control for Ag cross presentation; irradiated MMC cells were used as a specificity control for assessing Neu‐reactive T cell function. We then utilized a direct cytotoxicity assay to demonstrate that sorted Gr1^−/low^CD11b^−/low^ myeloid cells from tumor‐bearing mice boosted tumor‐reactive T cell‐mediated killing of MMC target cells, ex vivo (Fig. 7A, *P* = 0.001). These data suggest that although tumor burden drives the expansion of MDSCs and suppresses the expansion of mature Ly6G^+^ Ly6C^−^ APCs, these Gr1^−/low^CD11b^−/low^ cells retain their immune stimulatory function, but may not become fully functional in the presence of a weak antitumor immune response.

## 4 DISCUSSION

Here, we describe a new class of APC, Gr1^−/low^CD11b^−/low^ cells that do not express pan markers of myeloid DCs (CD11c), plasmacytoid DCs (Ly6C) or macrophage (F4/80). Characterization of these cells demonstrated their expression of MHCII and the costimulatory molecules CD80, CD86, and CD40 at the steady state. Further characterization of this population revealed that while the majority of Gr1^−/low^CD11b^−/low^ myeloid cells do not express T or B cell lineage markers, we found that B cells interact with this myeloid APC population, a phenomenon that has classically been described to occur between DCs and B cells.^35^, ^36^ This interaction may contribute to the immune stimulatory function of these atypical APCs, as such an interaction has been reported to boost immune stimulatory function of conventional DCs.^37^ The frequency of Fsc^hi^ Ssc^hi^ Gr1^−/low^CD11b^−/low^ APCs was significantly greater than that of DCs in the spleen. Importantly, Gr1^−/low^CD11b^−/low^ APCs were also present at the tumor site of animals bearing lung metastases at a frequency that was inversely proportional to that of MDSCs. Interestingly AIT drove the accumulation of Gr1^−/low^CD11b^−/low^ APCs while concomitantly reducing the frequency of Gr1^+^CD11b^+^ MDSCs both in the spleen and within the tumor bed; this was associated with an improved survival of tumor‐bearing animals.

Gr1^−/low^CD11b^−/low^ APCs were abundant in the steady state in naïve mice in vivo and had antitumor immune stimulatory function without any need for further ex vivo activation, although stimulation by LPS suggested they maintain the potential for further activation. These data suggest that Gr1^−/low^CD11b^−/low^ APCs may be optimal performers in terms of Ag uptake as well as Ag presentation. In fact, Gr1^−/low^CD11b^−/low^ myeloid cells were capable of Ag uptake and cross‐presentation with similar efficiency to CD11c+ DCs. This paradoxical property of Gr1^−/low^CD11b^−/low^ myeloid cells was associated with the presence of two subsets; a Ly6G^+^Ly6C^−^ subset and a Ly6G^−^Ly6C^−^ subset. While the Ly6G^+^Ly6C^−^ subset showed higher basal maturity, the emergence of which was associated with prolonged survival of tumor‐bearing mice, the Ly6G^−^Ly6C^−^ subset showed less maturity and higher responsiveness to LPS stimulation.

Tumor burden altered the frequency of Gr1^−/low^CD11b^−/low^ myeloid cells but did not impair their immune stimulatory function; these cells, when derived from either the control group or the AIT group, were able to boost tumor‐reactive T cell responses. Interestingly, AIT during tumor burden resulted in the modulation of the myeloid cell compartment, revealing an inverse relationship between Gr1^−/low^CD11b^−/low^ myeloid cells and MDSCs. Such modulation of Gr1^−/low^CD11b^−/low^ cells by AIT was associated with a significantly higher frequency of the Ly6G^+^Ly6C^−^ subset and splenic CD25+ NKT cells, which increased survival of animals. These observations are supported by previous work from our group and others.^23^, ^24^, ^27^, ^38^ It has previously been shown by our group that MDSCs can be rendered immune stimulatory in the presence of CD25^+^ NKT cells. The removal of NKT cells from tumor‐reactive lymphocytes resulted in the inability of AIT to modulate MDSCs to become immune stimulatory, and failed to protect animals from tumor challenge.^27^ Similar observations were made using PBMCs of patients with breast carcinoma showing that HER‐2/Neu‐specific T cell responses were sustained in the presence of MDSCs; these sustained T cell responses were associated with the loss of CD11b and the up‐regulation of HLA‐DR on MDSCs, as well as the presence of CD25+ NKT cells.^24^ Therefore, our current results suggest that a sufficient frequency of activated NKT cells in secondary lymphoid organs as well as the tumor microenvironment may modulate the myeloid cell compartment in tumor bearing mice to reduce the suppressive capacity of MDSCs, while also driving the emergence of Ly6G^+^Ly6C^−^Gr1^−/low^CD11b^−/low^ immune stimulatory APCs.

The immune stimulatory function of Gr1^−/low^CD11b^−/low^ APCs was also associated with the induction of specific tumor cell killing by Ag‐sensitized T cells. In fact, our data suggest that Gr1^−/low^CD11b^−/low^ myeloid cells function as APCs to process and cross‐present tumor Ags to tumor‐reactive T cells, resulting in the promotion of antitumor immune responses. This was further confirmed by showing a higher antitumor function of T cells in the presence of Gr1^−/low^CD11b^−/low^ myeloid cells, as well as the ability of these cells to uptake Ag, and to cross‐present to tumor‐reactive T cells. These properties of Gr1^−/low^CD11b^−/low^ APCs make them a potential candidate for a cell‐based immunotherapy of cancer without having limitations of DC‐based vaccines. Such impaired DC function is attributed to MDSCs both in vivo^39^ and in vitro.^40^ Similar MDSC‐mediated suppressive function of macrophages has been reported in cancer patients.^41^ Furthermore, DC‐intrinsic immune suppressive activity has been reported in cancer patients as well as in animal models of transplanted and spontaneous carcinoma.^42^, ^43^, ^44^, ^45^, ^46^

In summary, we have identified Gr1^−/low^CD11b^−/low^ myeloid cells that possess characteristics of APCs that are unique in the following ways: (i) they are more abundant than DCs, (ii) they are heterogeneous making them highly effective in both Ag uptake and Ag presentation simultaneously, (iii) they retain their immune stimulatory function during tumor burden, and are inversely correlated with MDSCs, and (iv) their frequency is increased in the presence of CD25+ NKT cells. Moreover, human CD33^+^CD11b^−/low^HLA‐DR^+^ myeloid cells appear to have similar immune stimulatory function as murine Gr1^−/low^CD11b^−/low^ APCs.^24^

We thank Julie Farnsworth and Qingzhao Zhang of Virginia Commonwealth University for their assistance with cell sorting. We also thank Dr. Jose R. Conejo‐Garcia for graciously providing key reagents.

## Supporting information

Supplemental Fig. 1
