# Supplementary material for: Gr1−/lowCD11b−/lowMHCII+ myeloid cells boost T cell anti‐tumor efficacy
Source: J Leukoc Biol. 2018 Jul 9;104(6):1215–28. doi: 10.1002/JLB.5A0717-276RR (PMC6258302; doi:10.1002/JLB.5A0717-276RR)
Supplement: Supplementary file 1 — Supplemental Fig. 1 [file JLB-104-1215-s001.pdf]

Supplemental Fig. 1

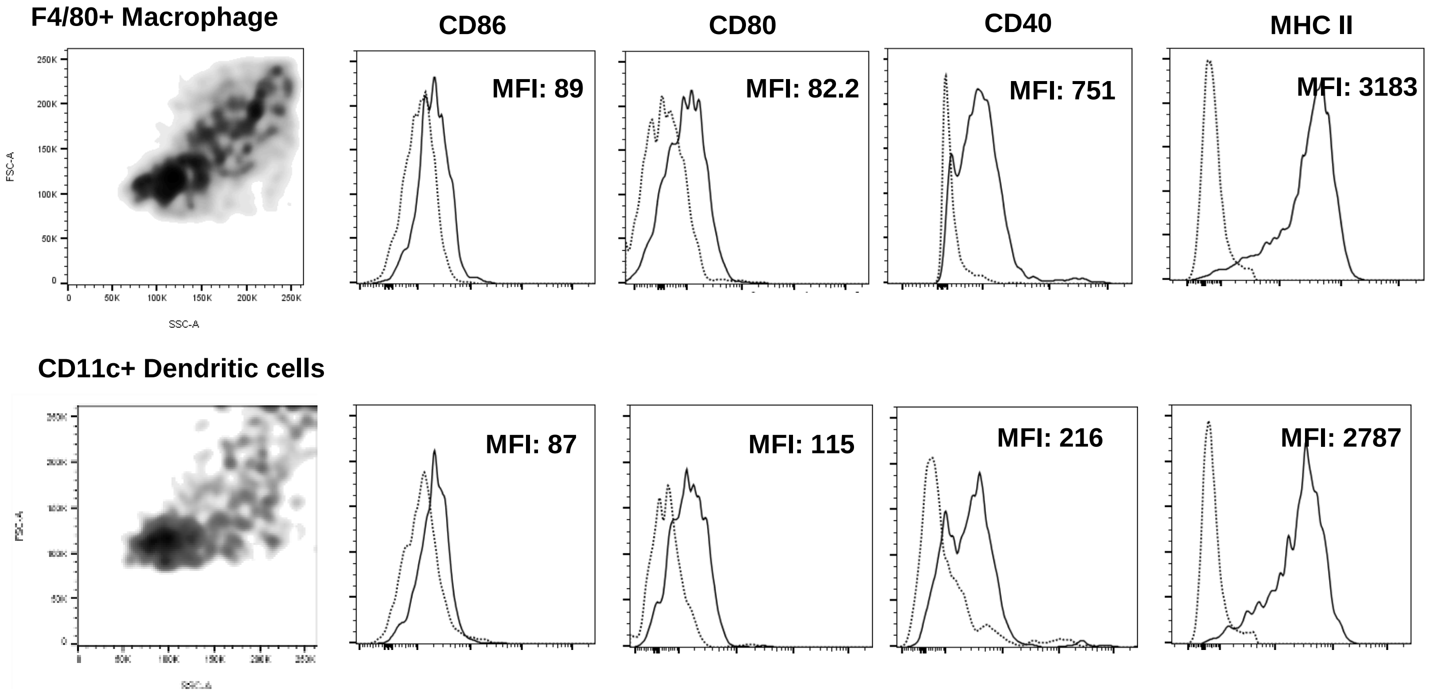

Supplemental Figure 1. Characterization of size, granularity, and expression of costimulatory molecules of naïve splenic macrophages and dendritic cells. Spleens harvested from FVBN202 mice were processed into a single cells suspension, and then subjected to a multicolor FACS analysis to assess the indicated parameters. MFI is reported after subtraction of the isotype control.

Supplemental Fig. 2

Supplemental Figure 2. Splenic Gr1$^{-/low}$CD11b$^{-/low}$ cells show monoblast-like and lymphocyte-like morphology. Sorted splenic Gr1$^{-/low}$CD11b$^{-/low}$ cells of naïve FVBN202 mice were analyzed by DiffQuick staining to determine their morphology.

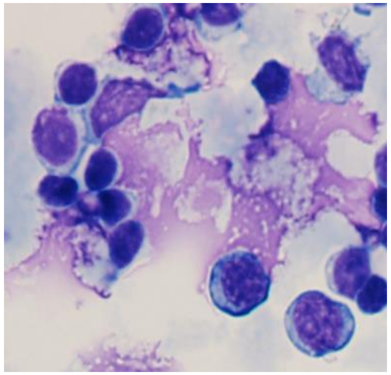

Supplemental Fig. 3

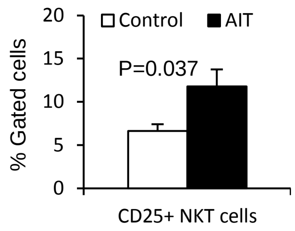

Supplemental Figure 3. AIT increases the frequency of activated NKT cells, *in vivo*. Splenocytes of FVBN202 mice bearing metastatic mammary tumor in the lung who remained untreated (Control) or received AIT (AIT) were collected for flow cytometry analysis. Gated CD4-CD8- cells were analyzed for CD49b+CD3+CD25+ NKT cells. Data represent mean ± SEM of 3 independent experiments.

Supplemental Fig. 4

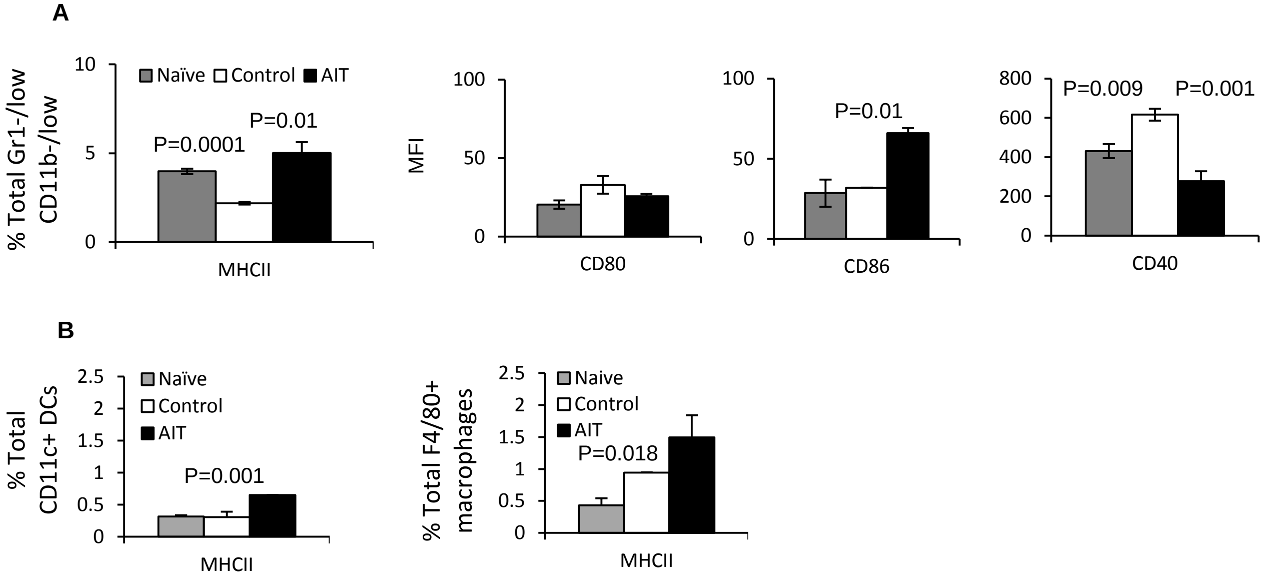

Supplemental Figure 4. Tumor burden changes Gr1$^{-/low}$CD11b$^{-/low}$ cells, and AIT restores them to the levels similar to those in naïve mice. A) Gated splenic Gr1$^{-/low}$CD11b$^{-/low}$MHCII^{+} cells of naïve FVBN202 mice (Naïve), tumor-bearing mice (Control) and tumor-bearing mice after AIT (AIT) were analyzed for their total frequency and expression of co-stimulatory molecules. B) Total frequency of splenic CD11c+CD11b+MHCII+ or F4/80+CD11b+MHCII+ cells. Data represent mean ± SEM of triplicate experiments.

Supplemental Fig. 5

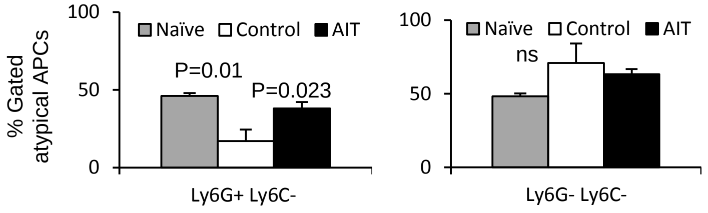

Supplemental Figure 5. Tumor burden changes frequency of two subsets of Gr1$^{-/low}$CD11b$^{-/low}$ APCs, and AIT restore them to the levels similar to those of naïve mice. Gated APCs of naïve FVBN202 mice (Naïve), tumor-bearing mice (Control) and tumor-bearing mice after AIT (AIT) were analyzed for the frequency of the Ly6G^{+}Ly6C^{-} and Ly6G^{-}Ly6C^{-} subsets. Data represent mean ± SEM of triplicate experiments.

Supplemental Fig. 6

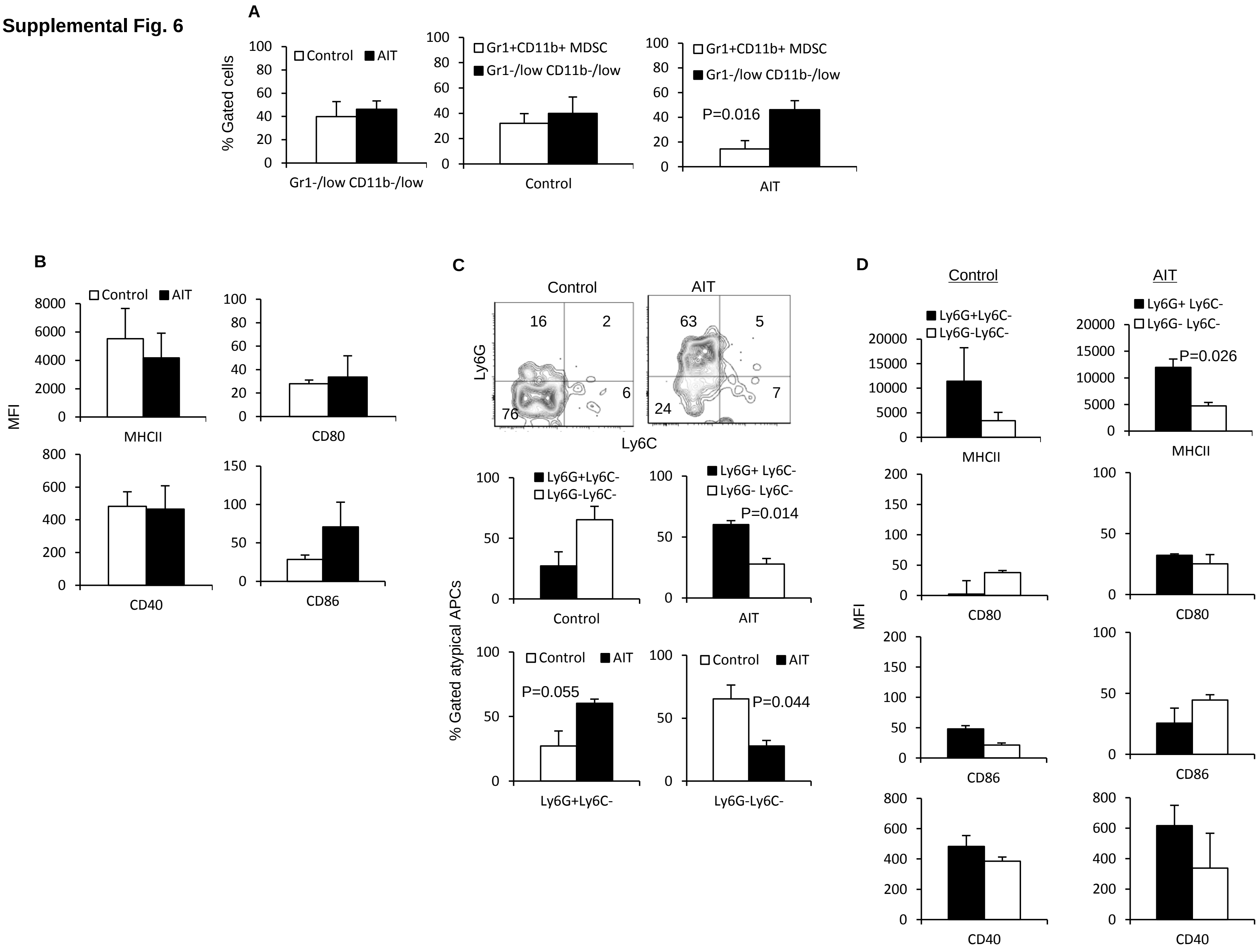

Supplemental Figure 6. Gr1$^{-/low}$CD11b$^{-/low}$ APCs accumulate within lung metastases upon AIT. FVBN202 mice (n=2) were injected with 10^5 MMC cells i.v. Animals were sacrificed when they became moribund, and lung metastases were harvested, digested, then subjected to analysis by flow cytometry. A) Frequencies of Gr1$^{-/low}$CD11b$^{-/low}$ cells and MDSCs in control or AIT-treated mice. B) Expression of MHC II and co-stimulatory molecules on gated Gr1-/low CD11b-/low cells in control or AIT-treated mice. C) Frequency of the Ly6G^{+}Ly6C^{-} - and the Ly6G^{-}Ly6C^{-} cells as gated on CD11b-MHCII+ cells in control or AIT-treated mice. D) Expression of MHCII and costimulatory molecules as gated on Ly6G^{+}Ly6C^{-} CD11b- MHCII+ or Ly6G^{-}Ly6C^{-} CD11b- MHCII+ cells in control or AIT-treated mice. Data represent mean ± SEM of duplicate experiments
